# Disruption of microtubule function in cultured human cells by a cytotoxic ruthenium(ii) polypyridyl complex†

**DOI:** 10.1039/c9sc05671h

**Published:** 2019-11-18

**Authors:** Nagham Alatrash, Faiza H. Issa, Nada S. Bawazir, Savannah J. West, Kathleen E. Van Manen-Brush, Charles P. Shelor, Adam S. Dayoub, Kenneth A. Myers, Christopher Janetopoulos, Edwin A. Lewis, Frederick M. MacDonnell

**Affiliations:** Department of Chemistry and Biochemistry, University of Texas at Arlington Arlington TX 76019 USA macdonn@uta.edu; Department of Biological Sciences, University of the Sciences Philadelphia PA 19104 USA; Department of Chemistry, Mississippi State University Starkville MS 39762 USA

## Abstract

Treatment of malignant and non-malignant cultured human cell lines with a cytotoxic IC_50_ dose of ∼2 μM tris(4,7-diphenyl-1,10-phenanthroline)ruthenium(ii) chloride (**RPC2**) retards or arrests microtubule motion as tracked by visualizing fluorescently-tagged microtubule plus end-tracking proteins. Immunofluorescent microscopic images of the microtubules in fixed cells show substantial changes to cellular microtubule network and to overall cell morphology upon treatment with **RPC2**. Flow cytometry with MCF7 and H358 cells reveals only minor elevations of the number of cells in G_2_/M phase, suggesting that the observed cytotoxicity is not tied to mitotic arrest. *In vitro* studies with purified tubulin reveal that **RPC2** acts to promote tubulin polymerization and when imaged by electron microscopy, these microtubules look normal in appearance. Isothermal titration calorimetry measurements show an associative binding constant of 4.8 × 10^6^ M^−1^ for **RPC2** to preformed microtubules and support a 1 : 1 **RPC2** to tubulin dimer stoichiometry. Competition experiments show **RPC2** does not compete for the taxane binding site. Consistent with this tight binding, over 80% of the ruthenium in treated cells is co-localized with the cytoskeletal proteins. These data support **RPC2** acting as an *in vivo* microtubule stabilizing agent and sharing many similarities with cells treated with paclitaxel.

## Abstract 2

Treatment of cultured human cell lines with a cytotoxic IC_50_ dose of ∼2 μM tris(diphenylphenanthroline)ruthenium(ii) chloride (**RPC2**) retards or arrests microtubule motion as tracked by visualizing fluorescently-tagged microtubule plus end-tracking proteins.

## Graphical abstract

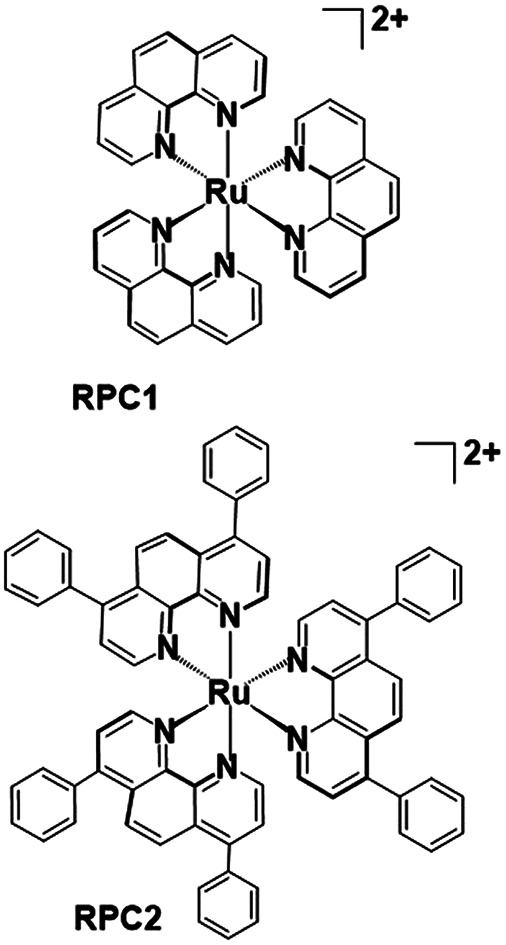

## Introduction

Microtubules (MTs) play an essential role in mitosis, cellular structure, and trafficking, as well as offer a promising target for innovative chemotherapeutic agents.^1,2^ MTs are composed of αβ-tubulin heterodimers that undergo highly regulated and dynamic bouts of polymerization and depolymerization. Microtubule targeting drugs disrupt this ‘dynamic instability’ by inhibiting or promoting polymerization and interfere with mitosis and other essential cellular processes, leading to apoptosis.^3,4^ Agents that inhibit tubulin polymerization, such as nocodazole (NCZ), vincristine, and colchicine, are known as microtubule destabilizing agents (MDAs) and are used therapeutically.^5,6^ Microtubule stabilizing agents (MSAs), more recently discovered, inhibit MT depolymerization. The first identified MSA, paclitaxel (PTX, Taxol), was discovered in 1979,^7^ and approved for clinical use in 1993 for treatment of solid tumor malignancies.^4,8–10^ Since this time a number of synthetic derivatives, *e.g.* docetaxel, or new types of MSAs, such as epothilones, laulimalide, discodermolide, cetamine A and B, dictyostatin, peloruside A, dactylolide, and zampanolide, have been discovered; most of which are natural products believed to have evolved as broad-spectrum toxins to target MTs in prey or predators.^8,11^ These MSAs are complex organic molecules with multiple fused ring structures and chiral centers in which the regio and stereochemistry must be controlled. Their large-scale synthesis is often a major undertaking and an important consideration in their development.

**Fig. 1:**
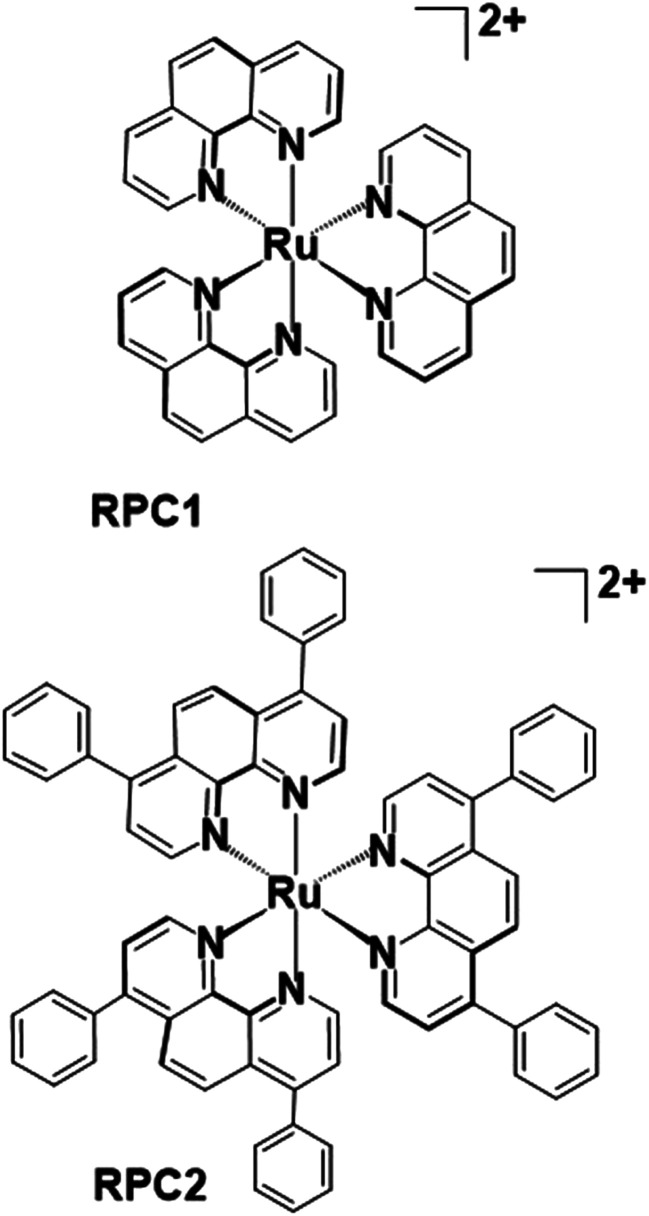
Chemical structure of tris(1,10-phenanthroline)ruthenium(ii) (**RPC1**) and tris(4,7-diphenyl-1,10-phenanthroline)ruthenium(ii) (**RPC2**) cations. These ruthenium(ii) polypyridyl complexes are used as the chloride salts in this study.

The only reports of metal complexes targeting microtubules in cells are limited to the activity of simple ions, such as As^3+^, Pb^2+^, and Hg^2+^,^12–14^ and one organometallic compound, Os_3_(CO)_10_(NCMe)_2_.^15^ The simple ions generally destabilize MTs whereas the trinuclear osmium carbonyl cluster is proposed to lose the two labile MeCN and react with tubulin sulfhydryl groups to induce hyperstabilization of the MT structure. Simple and ubiquitous ions like Mg^2+^ and Ca^2+^ are required for MT polymerization and depolymerization^16–18^ and at elevated, usually non-physiologically relevant, concentrations of these cations cause MT depolymerization.^19^ We and others have reported on the potent cytotoxicity of [Ru(DIP)_3_]Cl_2_ (Fig. 1) in numerous malignant (H358, MCF7, CCL228, HL60, B16, MDA-MB-231, A549, Jurkat, ML2, SF) and non-malignant (MCF10a) cell lines.^20–23^ IC_50_'s consistently range between 1 and 4 μM irrespective of the cell type. Conversely, the structurally similar but smaller [Ru(phen)_3_]Cl_2_ (Fig. 1) rarely shows any cytotoxicity below 50 μM.^20^ Herein, we show that these two coordination complexes promote tubulin polymerization *in vitro*, and, more significantly, **RPC2** binds MTs *in vivo* (live cells) and induces massive changes to the MT structure and dynamics. This MT disrupting activity correlates with the observed cytotoxicity and supports this as the dominant apoptotic mechanism of action.

## Experimental

### Materials and methods

#### Reagents

Ruthenium(iii) chloride trihydrate (Pressure Chemical Co) was used as received, tetrabutyl ammonium chloride hydrate, 4,7-diphenyl-1,10-phenanthroline (DIP), 1,10-phenanthroline (phen), ammonium hexafluorophosphate, ethanol, acetonitrile (Aldrich) were used as received. [Ru(1,10-phenanthroline)_3_]^2+^ (**RPC1**) was synthesized according to literature.^24^ PTX, tubulin porcine brain (>99% pure), general tubulin buffer and glycerol tubulin buffer were purchased from Cytoskeleton Inc., (Denver. CO). NCZ was purchased from Sigma Aldrich. The complete EDTA-free Protease Inhibitor Cocktail Tablets was purchased from Roche.

#### Synthesis of [Ru(DIP)_3_]Cl_2_ (**RPC2**)

This complex was prepared by using a modified literature procedure.^24,25^ DIP (5.6 g, 17 mmol) and RuCl_3_·*x*H_2_O (38% Ru by mass) (0.74 g, 2.8 mmol) were suspended in 150 mL of ethanol. After refluxing overnight, the mixture was cooled to room temperature, filtered, and the volume reduced to 50 mL by rotary evaporation. The product was precipitated by adding an aqueous ammonium hexafluorophosphate (NH_4_PF_6_) dropwise until a precipitate was clearly present. The precipitate was filtered and washed with ethanol followed by washing with copious amount of water and dried *in vacuo* at 60 °C for 12 h. Yield 3.0 g of the hexafluorophosphate salt (83%). The NMR data are identical to that reported in the literature ^1^H NMR (CD_3_CN) *δ* = 7.59–7.62 (m, 30H, H_DIP_), 7.64 (d, 6H, *J*_HH_ = 5.7 Hz, H_3_, H_8_), 8.20 (s, 6H, H_5_, H_6_), 8.25 (d, 6H, *J*_HH_ = 5.1 Hz, H_2_, H_9_).

The chloride salt was prepared from the hexafluorophosphate salt by dropwise addition of a concentrated solution of tetra-*n*-butylammonium chloride in acetone to a concentrated solution of the [Ru(DIP)_3_][PF_6_]_2_ salt in acetone. The resulting precipitate was filtered and washed with acetone, then dried *in vacuo* at 60 °C. Yield 2.25 g (95%) [Ru(DIP)_3_]Cl_2_.

#### Preparing **RPC2** aqueous solutions

**RPC2** (chloride salt) is sparing soluble in water or buffer unless it is first dissolved in a small amount of DMSO and then diluted into the aqueous solution. All **RPC2** solutions in this work were prepared in this manner and contain 5% or less DMSO by volume. Vehicle only solutions contain the same amount of DMSO as the **RPC2** solutions. When prepared this way, **RPC2** solutions readily passed through a nylon filter (0.2 μM pores), and showed no light scattering (Tyndall effect). In order to avoid precipitation, freshly prepared solutions of **RPC2** were used in all cases.

#### Tubulin polymerization assay

Tubulin polymerization was determined according to the manufacture's protocol by a biochemical Optical Density-based assay (Cytoskeleton, Inc., CO). Briefly, tubulin porcine brain (>99% pure Cat# T240-DX) was reconstituted with ice-cold buffer (80 mM PIPES pH 6.9, 2.0 mM MgCl_2_, 0.5 mM EGTA, with 10 μL of 100 mM GTP and kept on ice). Tubulin stock solutions (10 mg mL^−1^) were aliquoted into 5 × 200 labeled tubes and immediately drop-frozen in liquid nitrogen and stored at −70 °C. Polymerization was started by adding 100 μL volume of 3 mg mL^−1^ tubulin in 80 mM PIPES pH 6.9, 0.5 mM EGTA, 2 mM MgCl_2_, 1 mM GTP, 10% glycerol buffer to pre-warmed 96 well half area plate at 37 °C, and followed by absorbance readings at 340 nm over 60 minutes at 37 °C (temperature-controlled microtiter plate reader). Absorbance at 340 nm was determined using FLUO-star Omega microplate reader (BMG Labtech) in kinetic absorbance mode. Polymerization reactions included control (control minus ligand or anti-cancer agent vehicle), and 10 μM of PTX, NCZ, **RPC1** and **RPC2**. OD is proportional to the concentration to the polymerized tubulin. The experiment was performed in triplicate (mean values are presented). Data were exported and polymerization plotted using Excel software. At concentrations of 1 μM or greater, **RPC2** has an appreciable absorbance at 340 nm, therefore this was measured and subtracted to normalize the data.

#### Electron microscopy

Aliquots of polymerized tubulin were placed on 300-mesh carbon-coated, Formvar-treated copper grids and immediately stained with 5–10 successively applied drops of 1% (w/v) uranyl acetate. Excess stain was wicked from the grids with torn filter paper. The grids were examined in a Hitachi H-9500 High-resolution Transmission Electron Microscope.

#### Immunoblotting

Cells are treated with drugs for 4 h in 37 °C incubator. The confluent 60 mm dishes of cells were washed 3 times with 37 °C PBS, then lysed in microtubule stabilization buffer (Tris–HCl pH 6.8, 20 mM, NaCl 140 mM, MgCl_2_ 1 mM, NP40 0.5%, EGTA 2 mM, 10 μM of Taxol) and protease inhibitors (100× Protease Inhibitor Cocktail (PIC) and 100× phenylmethane sulfonyl fluoride (PMSF)). Then, the cells were scraped, mixed and quickly transfer to microtubes. These samples were then spun at 10 000 rpm for 20 minutes, the supernatants containing free tubulin were collected and a bicinchoninic acid (BCA) protein assay was performed to check the protein quantity. Equal amounts of protein sample were prepared in Laemmli sample buffer and were boiled for 5 min at 95 °C. Proteins were resolved on a 12% polyacrylamide gel. The proteins were transferred to a nitrocellulose membrane for 1.5 h at 100 V. The nitrocellulose membrane was stained for 1 min with Ponceau Red to verify the efficiency of the transfer. Then, the membrane was blocked in 5% skim milk for 1 h at room temperature and incubated with primary alpha tubulin monoclonal antibody in 5% BSA overnight at 4 °C. The membrane was washed 3 times with TBST buffer before being probed with secondary antibodies conjugated to HRP, then the membrane was incubated for 1 h at room temperature before it was washed 3 times again with TBST buffer. Protein bands were visualized with an enhanced chemiluminescent (ECL) system.

#### Isothermal titration calorimetry

ITC experiments were performed using a Microcal VP-ITC (Malvern) instrument. For polymerized porcine brain tubulin the calorimeter was set at 37 °C. For depolymerized porcine brain tubulin the calorimeter was set at 4 °C. Tubulin concentration was kept below 3 mg mL^−1^ to ensure that minimal microtubule formation before any polymerization assays were performed in preparation for ITC experiments. Reverse titrations were used in all ITC experiments due to the low solubility of **RPC2** in aqueous solutions. A typical ITC experiment involved fourteen 20 μL injections of tubulin (∼30 μM heterodimers, or 2 mg mL^−1^) into a 1.45 mL cell of ligand solution. The ITC thermograms were corrected for titrate and titrant dilution effects by performing the appropriate blank experiments and correcting the observed heats by subtracting the heats of dilution. Corrected ITC titrations were fit with a nonlinear regression algorithm using CHASM, an ITC data analysis program developed in the Lewis laboratory to determine the thermodynamic parameters, including the association constant (*K*) and changes in free energy (Δ*G*), enthalpy (Δ*H*), and entropy (−*T*Δ*S*).

#### Subcellular localization assay

H358 cell line was seeded in 60 × 60 mm dishes and grown to 80% confluency not to exceed 5 × 10^6^ cell density. The cell line was then treated with 20 μM concentrations of **RPC1** or **RPC2** for 12 h. The cells were washed, trypsinized, and centrifuged for 5 min at 1000 g to create cell pellets. Pellets were washed 3 times with ice cold phosphate buffer saline (PBS). The cell pellet was then fractionated using a Qproteome Cell Compartment Kit (Qiagen, Germany) following the procedure outlined in the associated handbook, dated October 2012. Four protein fractions were obtained: cytosolic proteins, membrane proteins, nuclear proteins, and cytoskeletal proteins. The mitochondria, Golgi apparatus, and endoplasmic reticulum are isolated in the membrane protein fraction. Each fraction was diluted to a total volume of 5.00 mL with a solution of 1% HNO_3_ water in ultrapure water. Solutions were analyzed for ruthenium ion concentration using ICP-MS.

#### Indirect immunofluorescence analysis

Cells were cultured on coverslips and placed in individual wells of six-well plates. After overnight incubation, cells were treated with drugs for 12 h fixed with 4% paraformaldehyde. Fixed cells were permeabilized using 0.25% Triton X-100 in PBS, followed by blocking with 5% BSA, then probed with alpha-tubulin antibody and incubated over night at 4 °C. Next day, Alexa Fluor 488-conjugated goat anti-mouse IgG (H + L) secondary antibody was administered for 2 h at room temperature followed by staining the nucleus with propidium iodide (PI) for 5 minutes at room temperature. The immunolabeled coverslips were mounted on glass slides. Microscopic images were obtained using Zeiss LSM 510 with 63× oil objective. PI (*λ*_ex_ = 514 nm) and Alexa Fluor 488-conjugated (*λ*_ex_ = 488 nm) were collected at 530–617 and 505–530 nm respectively.

#### Cell cycle analysis

Flow cytometric analysis of cellular DNA content was obtained using a Propidium Iodide Flow Cytometry Kit (ab139418, Abcam). MCF7 and H358 cell line were seeded in 60 × 60 mm dishes and grown to 80% confluency not to exceed 6 × 10^5^ cell density. The cell lines were then treated with **RPC2** for 12 h at 37 °C. The cells were trypsinized and centrifuged at 500 g for 5 min, washed with PBS, fixed in 66% ice cold ethanol at 4 °C for 2 h. Fixed cells were centrifuged at 500 g for 5 min and washed with PBS and resuspended in 200 μL of PBS containing 50 μg mL^−1^ PI and 550 U mL^−1^ RNase for 30 min at 37 °C, and subjected to flow cytometry (BD LSR II flow cytometer). DNA histograms were analyzed using DIVA software.

#### Spinning disk confocal imaging and image processing

MCF7 cells were cultured in phenol-red DMEM media (HyClone™, GE healthcare) supplemented with 10% FBS and penicillin/streptomycin at 37 °C in 5% CO2. MCF10A cells were cultured in low-calcium phenol-red DMEM/F12 (HyClone™, GE healthcare) supplemented with final concentration of 5% horse serum, 20 ng mL^−1^ EGF, 0.5 mg mL^−1^ hydrocortisone, 100 ng mL^−1^ Cholera toxin, 10 μg mL^−1^ insulin, and 1% pen-strep. For live imaging, 500 000 cells per mL of both MCF7 and MCF10A cells were transfected with the cDNA (EGFP-EB3), and then cultured on 10  μg mL^−1^ fibronectin coated 35 mm glass-bottom dishes (Cellvis, cat#: D35-20-1.5-N). Transfection of EGFP-EB3 was performed using a Lonza Nucleofector Device with Ingenio electroporation kit (Cat# MIR 50112, Mirus). Cells were treated with **RPC2** 18–24 hours post-transfection to allow time for cDNA expression. To minimize photobleaching, phenol-free DMEM was supplemented with 10% FBS and 25 mM  HEPES, pH = 7.2 replaced the original media and was administered before **RPC2** treatment. Time-lapse imaging was obtained before, 30 min, 1 h, 2 h, and 3 h after **RPC2** treatment for both MCF7 and MCF10A. Images were obtained on a Zeiss Axiovert (Jena, Germany) microscope with a Zeiss alpha Plan-Fluar 100×, 1.45 NA oil objective and a spinning disk confocal scan head (Yokogawa CSU-X1, Tokyo, Japan). A 488 nm laser source with 488/25 nm, excitation filter was used for the illumination of GFP. Digital images were captured sequentially by a high speed EM-CCD camera (Photometrics Evolve™ 512, Tucson, AZ). Time-lapse experiments were automated by software (Slidebook 6.0.9, 3i, Denver, CO).

## Results

### General properties of ruthenium polypyridyl complexes (RPCs)

While there are numerous types of ruthenium polypyridyl complexes, one of the most commonly explored subtypes are the tris-chelate complexes which are coordinatively saturated and kinetically inert. **RPC1** and **RPC2** are two such complexes which exhibit exceptional chemical stability and which appear to be metabolically stable as rats and mice given intraperitoneal injections of radiolabeled [^106^Ru(phen)_3_][ClO_4_]_2_ excrete the intact cation in the urine in about 12 h.^26^ Additional examples of this exceptional stability include surviving in boiling concentrated acids or alkalis,^27^ or the use of **RPC1** as a digestive marker for ruminant animals since it is not absorbed by the intestine and passes through the animal.^27–29^ This chemical robustness is one of the reasons that RPCs are so extensively studied as biological probes or therapeutics.^30–32^ In general, the complex should be considered as robust as the free ligands excepting when irradiated which can lead to ligand loss.^33,34^ Importantly, the tris chelate RPCs, such as **RPC1** and **RPC2**, are chiral (propeller molecules with *D*_3_ point group symmetry) and are generally isolated as a racemic mixture of Λ and Δ enantiomers. No attempt to work with enantiopure complexes was made in this work.

### **RPC2** co-localizes with the cytoskeletal proteins

**Fig. 2:**
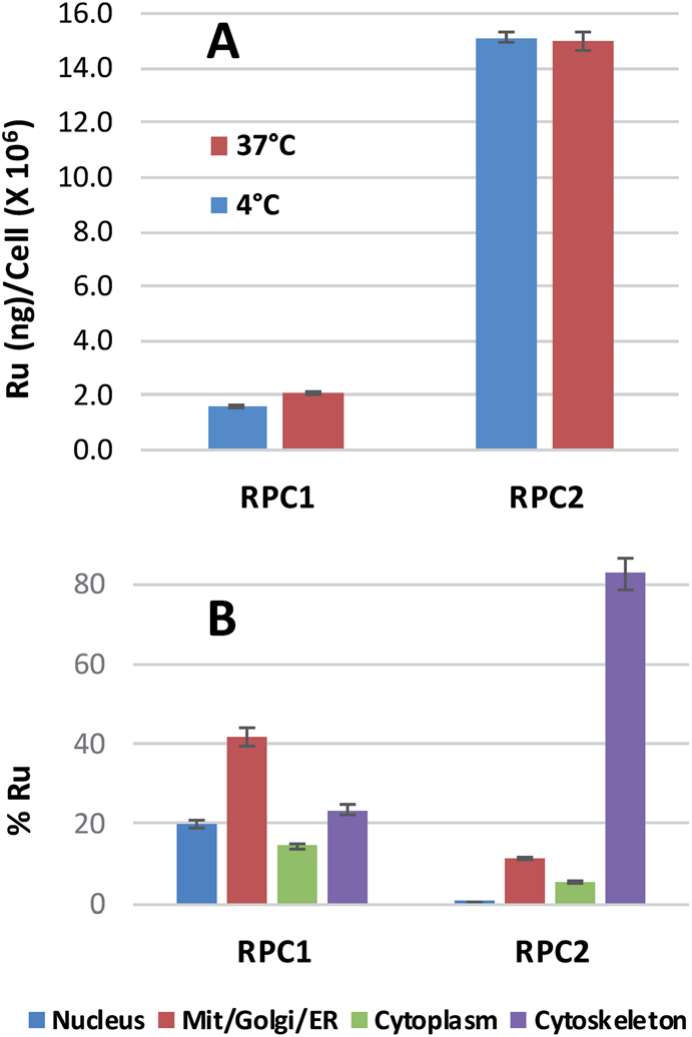
Ru content in whole (top) and fractionated (bottom) H358 cells. (A) Mass of ruthenium in ng per million cells for H358 cells incubated with 20 μM of **RPC1** or **RPC2** for 12 h at 37 °C or 4 °C. (B) Percent ruthenium found in four different fractions of H358 cells (nucleus, cytosol, mito/Golgi/ER, cytoskeleton). The cells were fractionated using a QIAGEN Compartment Kit and Ru ion content was analyzed using ICP-MS. The total amount of RPC 1 in the fractionated cells was 2.0 ng per million cells and **RPC2** 15 ng per million cells.

Ruthenium uptake by H358 cells incubated with 20 μM of **RPC1** or **RPC2** for 12 h at 37 °C reveals 2 ng Ru per million cells for **RPC1** and 15 ng Ru per million cells for **RPC2** (Fig. 2A). Additional studies were performed at 4 °C and gave similar uptake, supporting passive transport^35^ as the primary pathway for **RPC1** and **RPC2** entering cells (Fig. 2B). Passive diffusion through the cell membrane has been shown for the closely related RPC, [Ru(DIP)_2_dppz]^2+^ (dppz = dipyridophenazine), in HeLa cells, and appears to be a common transport mechanism for this class of compounds.^23,35–37^ The ruthenium uptake for [Ru(DIP)_2_dppz]^2+^ was reported to range between 2 to 50 ng Ru per million cells, depending on the incubation media. This is an excellent agreement with our data considering the cell line differences. Assuming an average cell volume of 1.7 pL (ref. 38) and homogenous distribution in the cell, intracellular ruthenium concentrations range from 16 to 400 μM for [Ru(DIP)_2_dppz]^2+^, ∼15 μM for **RPC1** and ∼112 μM for **RPC2**.

In order to assess the intracellular Ru distribution, treated H358 cells were fractionated into four components: nucleus, cytosol, membrane proteins, and cytoskeletal proteins. As shown in Fig. 2B, the biggest discovery was that over 80% of the Ru was found in the cytoskeletal fraction for cells treated with **RPC2**. To the best of our knowledge, there are no known interactions between RPCs and the cytoskeleton. Moreover, this heavily skewed distribution is not seen for **RPC1**, indicating selective binding between **RPC2** and some cytoskeletal proteins.

### **RPC2** induces changes in cellular morphology and MT structure in fixed cells

As shown in Table 1, the two RPCs under study have substantially differing levels of cytotoxic activity. **RPC1** is modestly cytotoxic with IC_50_ values are greater than 50 μM for the two malignant (MCF7, H358) and two non-malignant (MCF10, HUVEC) cell lines examined.

**Table 1:** The cytotoxicity IC_50_ of **RPC1**, **RPC2**, PTX, and NCZ against various cell lines

Cell line	Compounds IC_50_ (μM)			
	**RPC1**	**RPC2**	NCZ	PTX
H358	86.7 ± 4.1^a^	1.7 ± 0.1	1.0	0.1^b^
MCF7	>50	1.5 ± 0.3	3.2	0.025^c^
MCF10	>50	1.5 ± 0.5	0.48^d^	0.2^c^
HUVEC	92	2.8 ± 5.1	0.2^d^	8.2 × 10^−4^^e^

a Ref. 39.

b Ref. 40.

c Ref. 41.

d Ref. 42.

e Ref. 43.

In contrast, **RPC2** showed high cytotoxicity with low micromolar IC_50_ values against all four cell lines. For comparison, the IC_50_ values for nocodazole and paclitaxel are included in Table 1, both show low micromolar to nanomolar cytotoxicity.

**Fig. 3:**
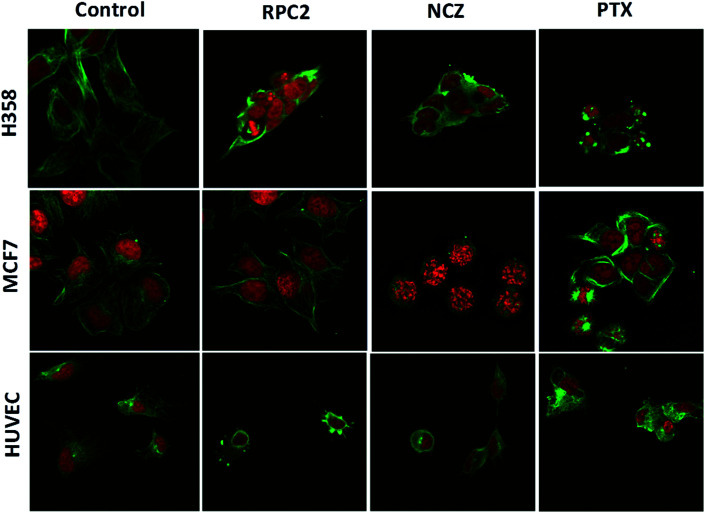
Fluorescent microscopic images of fixed cells immunostained for tubulin (green) and the nucleus (propidium iodide, red) after treatment with **RPC2**, NCZ, or PTX for 12 h. First row: H358 cells treated without (control), and with 1.7 μM **RPC2**, 1.0 μM NCZ, 0.1 μM PTX. Second row: MCF7 cells treated without (control), and with the 1.5 μM **RPC2** for 12 h, 3.2 μM NCZ, and 0.025 μM PTX for 12 h. Third row: HUVEC cells treated without (control), and with 2.8 μM **RPC2**, 0.2 μM NCZ, and 0.82 nM PTX for 12 h. Concentrations used were the IC_50_ for that cell line. The α-tubulin antibody was coupled with Alexa Fluor 488-conjugated goat anti-mouse igG (H + L) secondary antibody.

To determine if **RPC2** affected the cytoskeleton in cells, MTs were examined in MCF7, H358, and HUVEC using immunofluorescent labeling techniques and confocal fluorescent microscopy. The control cells (vehicle) showed that the MT structure is comprised of fine filaments spread throughout the cytoplasm (Fig. 3, first column). In these images, the MT's (anti-tubulin) are shown in green and the cell nucleus in red (propidium iodide, PI). Cells treated with the IC_50_ dose of **RPC2** for 12 h revealed a unique response to treatment (Fig. 3, second column). The H358 cells show dramatic changes with a collapse of the cell around the nucleus. The MTs became less organized and more condensed, and several cells displayed pyknotic nuclei, indicative of apoptosis. The MCF7 cells reveal little change in cell structure at this dose and time interval, although several nuclei appear in the early stages of DNA condensation. The HUVEC cells show large amounts of MT condensation, which appeared as Furinfoci or blobs at the edge of the cell membrane.

We compared these changes with those for known MDAs and MSAs and examined the cell's appearance after treatment with NCZ (IC_50_ dose) and PTX (IC_50_ dose), respectively. In the third column of Fig. 3, all three cell types became pyknotic upon treatment with NCZ, although each appeared in a different stage of nuclear degradation, indicating different temporal responses to the drug. Notably, there was not much MT structure evident, nor any bundles of MTs. PTX treatment also resulted in apoptotic cells; however, the MTs were clearly observed as bundles (MCF7 and H358) or as rounded blobs (HUVEC and H358; Fig. 3). In addition, several H358 and MCF7 cells showed extensive nuclear fragmentation indicating apoptosis (Fig. 3, second column). The MCF7 cells displayed elegant examples of MT bundles, organized at specific microtubule organizing centers in the cell, but somewhat abnormal as the bundles were thick and not dispersed evenly throughout the cell. Some cells were apoptotic with fragmented and condensed nuclei. The nuclei showed MT asters, including one in apparent metaphase, although oddly, the nuclear material appeared to be at the edge of the cell. The HUVEC cells treated with PTX showed the least perturbation from the control, but were nonetheless augmented.

**Fig. 4:**
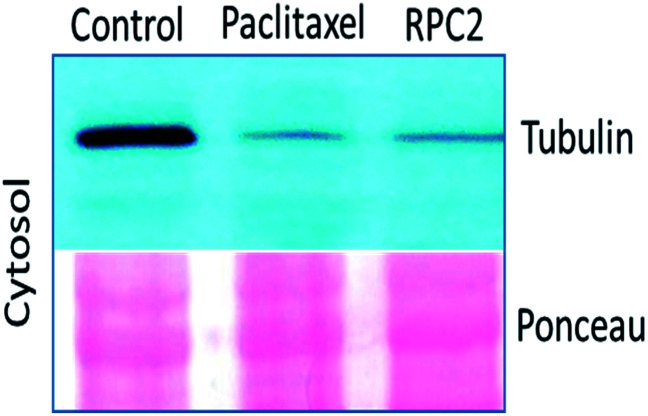
Western blot analysis of microtubule polymers (a-tubulin) isolated from H358 cells treated with the IC_50_ of PTX (0.1 μM), and **RPC2** (1.7 μM) for 12 h. The Ponceau staining shows equal protein loading.

Comparison of cells treated with **RPC2** with those treated with NCZ or PTX is not straightforward as some changes resemble traits exhibited by NCZ and others, PTX treated cells. Clearly there were differences in changes with cell type, with H358 showing the most dramatic variations with all three drugs. MCF7 responds strongly to NCZ and PTX, but not to **RPC2**. HUVEC cells responded most strongly to **RPC2** then NCZ and lastly PTX (Fig. 3). The formation of large foci of MTs (green blobs) in the H358 and HUVEC lines for **RPC2** suggests the compound is acting to condense and stabilize MTs. A western blot experiment analyzing the tubulin content in the cytosol of H358 cells treated with PTX (0.1 μM) or **RPC2** (1.7 μM) for 12 h before harvesting and lysis, shows that both agents act to substantially reduce the amount of free tubulin present the cytosol (see Fig. 4). This is interpreted as being due to the bulk of the free tubulin becoming sequestered in MTs due to the MSA activity.^44^

### **RPC2** inhibits MT assembly in live cells

To see how **RPC2** affects MT dynamics, live MCF7 and MCF10a cells were examined expressing a microtubule plus-end binding protein (end-binding protein 3; EB3) that is fused to a green fluorescent protein fragment (GFP-EB3) using spinning disk confocal fluorescent microscopy. Untreated cells revealed that the growing plus-ends of the MTs moved as green ‘comets’ demonstrating both a bright head and a lingering tail. A movie of these comets in an control cell shows the comets organized together in different parts of the cell, with groups of comets travelling in the same net direction or radiating out from various MT organizing centers (MOCs) at varying places, as others have shown previously (*e.g.* see Movie S1†).^45–48^

**Fig. 5:**
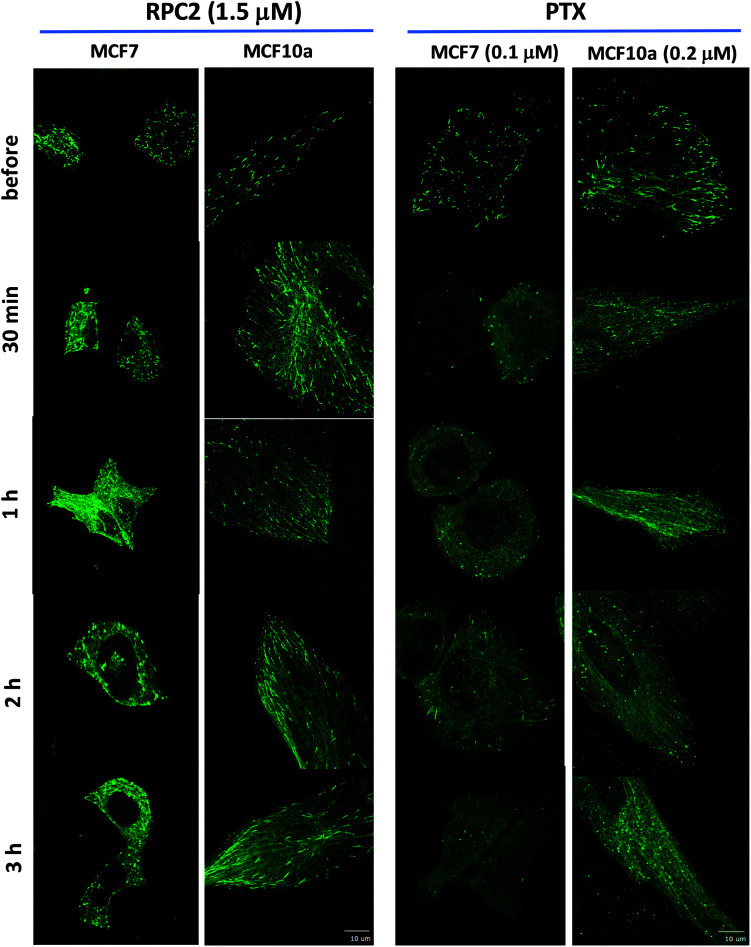
Spinning disk confocal fluorescent microscopic images of live MCF7 and MCF10a cells expressing the GFP-EB. Images show the initial frame of a short movie (4–9 s) and changes between images are due to the longer incubation period with 1.5 μM **RPC2** or 0.1 μM PTX for MCF7 and 0.2 μM PTX for MCF10a. A figure in which each frame is a clickable movie in the same format as given here is provided in the ESI (Fig. S1†).

Fig. 5 shows fluorescent images collected for control and RCF2-treated MCF7 and MCF10a cells at five time points (before treatment, 30 min, 1 h, 2 h, and 3 h post treatment) upon incubation with the IC_50_ dose of **RPC2** or PTX. Each image has a corresponding time-lapse movie, capturing at 3 to 10 s intervals, in which the movement or lack of movement of the comets was observed (see ESI Movies S1–S20,† all of which are embedded in ESI Fig. S1†).

Changes were apparent at the 1 h time point for cells treated with **RPC2** and 30 min for the PTX treated cells with effects for both more pronounced at longer time periods. For both **RPC2** and PTX, the comet's motion is retarded or stopped. The distinctive MT tracks are eventually lost and the signal disperses throughout the cytoplasm. In MCF7 cells treated with **RPC2**, the number of total comets decreases and those remaining become larger and less motile. Additionally, MT growth becomes disorganized with no clear net directional motion observed. These effects are more pronounced at longer time periods. MT function is better preserved in the MCF10a cells over the MCF7 cells, even at longer incubation times, suggesting less sensitivity to **RPC2** by MCF10a. However, this is not supported by the cytotoxicity data which has the IC_50_ for both lines at 1.5 μM. Presumably, this is an effect of the relatively short time period after introduction of the drug (3 h) compared to a typical MTT assay (96 h). PTX treated MCF10a cells are similarly more resistant to the effects of treatment over the MCF7 cells, but the level of MT disruption is greater here than seen with **RPC2**.

Some of the movies obtained with **RPC2** show strong photo-bleaching of the GFP signal with time. The movies of **RPC2**-treated MCF10a at 30 min, 2 h, and 3 h (Movies S7, S9 and S10), provide a typical examples of this effect. Initially, well-formed comets with tails are evident, but these barely move and simply fade to small round bright spots or disappear altogether as the movie progresses. Some of this bleaching is the result of the MTs growing slower, which increases the EB3 dwell time at the plus end of the MT. In addition, **RPC2** is also a well-known luminescent agent (*λ*_abs_ 460 nm; *λ*_em_ 640 nm)^49^ which makes it an excellent receptor for fluorescent resonance energy transfer (FRET) from the GFP excited state.^50^ Furthermore, **RPC2** is an excellent photosensitizer for the conversion of triplet oxygen to singlet oxygen, which can be highly destructive to any adjacent organic matter.^49^ Both likely happens with prolonged laser illumination used to obtain movies. The extent of the photobleaching varied and, in many instances, cells were imaged with little bleaching. To avoid this cell damage, we did not make multiple movies of any single cell and instead chose a different cell to image at the 30 min, 1 h, 2 h, and 3 h timepoints.

### **RPC2** treatment results in a small increase in cells in G_2_/M phase and a large increase in sub G_1_

**Fig. 6:**
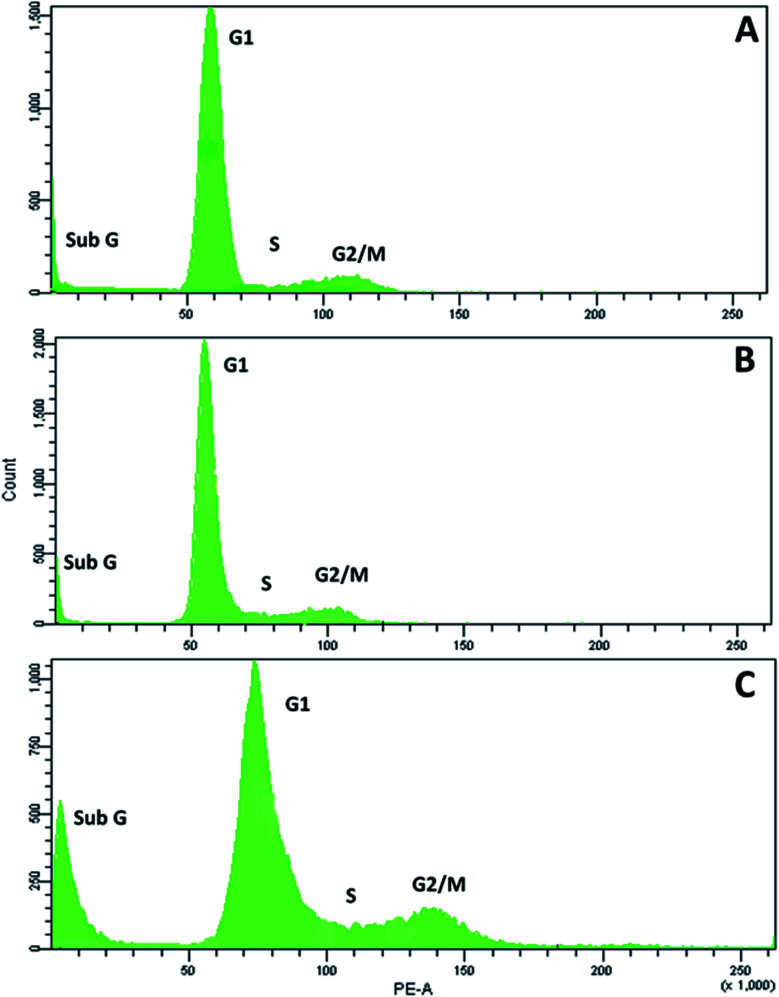
**RPC2** affects the cell cycle populations in MCF7 breast cancer cells. MCF7 cells were untreated (A) or treated with 1.5 μM (B) and 15 μM (C) of **RPC2** for 12 h, fixed with 66% ethanol, washed in PBS, treated with RNase for 30 min at 37 °C and stained with PI. Percent population: (A) sub G 7%, G1 78%, S 5%, G2/M 9%; (B) sub G 5%, G1 81%, S 6.3%, G2/M 7.8% (C) sub G 18%, G1 60%, S 8%, G2/M 14%.

Next, we examined the effects of **RPC2** on the cell cycle. As shown in Fig. 6, flow cytometry of MCF7 cells shows a small dose dependent response for cells treated with **RPC2**. At a 1.5 μM dose (the IC_50_), the histogram is essentially unchanged from the control whereas at the higher dose (15 μM), a clear jump in the percentage of cells in the sub G_1_ and G_2_/M phases is apparent. Populations of dead apoptotic or necrotic cells appear in the region prior to the G_1_ peak in flow cytometry, as the cell size has shrunk and DNA dye fluorescence is generally weakened with the loss of DNA structure.^51^ The percentage of cells in the G_2_/M phase rises from 9% to 14% while the percentage of sub G_1_ increased from 7% to 18%. Flow cytometry of H358 cells gives similar results, with increases of both the sub G_1_ and G_2_/M populations with a 10× dose of **RPC2** (see ESI Fig. S2†).

### **RPC2** act as microtubule stabilizing agents *in vitro*

**Fig. 7:**
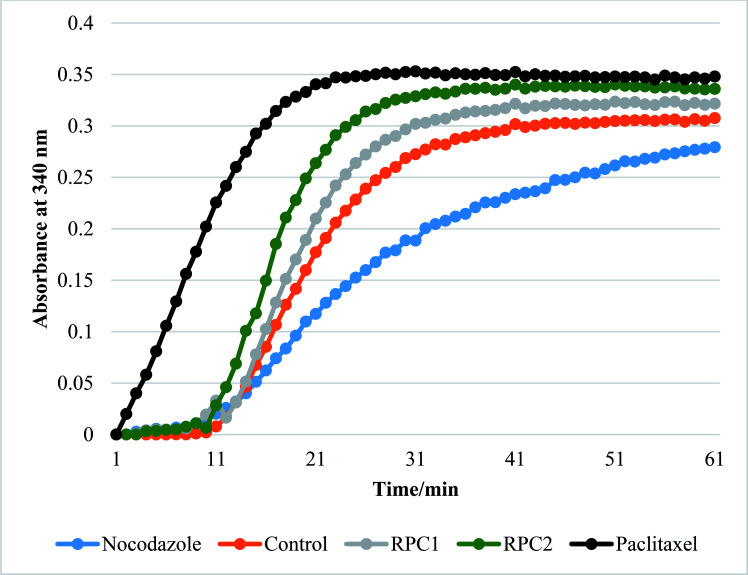
Effect of different ligands on tubulin polymerization *in vitro*. The change in turbidity measured by light transmission at 340 nm. Increasing turbidity indicated tubulin polymerization upon a temperature jump from 4 °C to 37 °C in the presence of 1 mM GTP and 10% glycerol in general tubulin buffer (80 mM PIPES pH 6.9, 2 mM MgCl_2,_ and 0.5 mM EGTA). All runs with added drug were done by addition of enough drug to make a 10 μM solution of drug with the tubulin (3 mg mL^−1^). The orange plot shows the normal tubulin polymerization growth curve in the absence of any drug. PTX (black plot) was used as a control to show how a microtubule stabilization agent effects polymerization. NCZ (blue plot) was added as control to show how a microtubule destabilizing agent effects polymerization. The legend on the bottom shows the color and markers for the **RPC1**, and **RPC2** treated runs. All plots are an average of three individual experiments. Error bars (±0.05 OD) omitted for clarity.

*In vitro* tubulin polymerization assays reveal that **RPC2** increases the rate of tubulin polymerization and enhances the extent of tubulin polymerization (see Fig. 7) relative to the no drug control. The effect is dose dependent (see ESI, Fig. S3†) and titrations between 0.1 and 50 μM **RPC2** reveal the onset of this effect at doses as low as 0.1 μM. Not only does **RPC2** increase the rate of tubulin polymerization relative to the control, it increases the overall degree of polymerization seen in solution at steady-state. Significantly, tubulin polymerization is also enhanced upon addition of **RPC1** (10 μM), although not to the same extent as with 10 μM **RPC2** (see Fig. 7). In this figure, the effects of PTX and NCZ on tubulin polymerization are included for comparison. The *in vitro* activity of **RPC1** suggests that RPCs in general may have some inherent MSA activity, however the lesser ability to enter the cells and the more even distribution throughout the cell (see Fig. 2) show this alone does not translate to cellular activity. Clearly, the DIP ligand increases the tubulin binding affinity and indicates that SAR studies can improve their performance. TEM images of the MTs formed in the presence of **RPC2** (10 μM) are unchanged in appearance from those of the control or those treated with PTX (10 μM), whereas only small MT fragments or tubulin dimers are apparent when treated with NCZ (see ESI Fig. S4†).

### **RPC2** binds preformed MTs with a binding affinity similar to that of DTX and does not compete for the DTX binding site

Isothermal titration calorimetry (ITC) was used to measure the binding thermodynamics of **RPC2** with tubulin and MTs. Reverse titrations (protein introduced into solutions with a fixed ligand concentration) were done as the poor solubility of **RPC2** limited its concentration range. For comparison, ITC measurements of colchicine and docetaxel (DTX) were also performed. The association constant (*K*), changes in free energy (Δ*G*), enthalpy (Δ*H*), and entropy (−*T*Δ*S*) were obtained^52,53^ are listed in Table 2 and were obtained by fitting a binding isotherm using the CHASM software to the raw integrated isotherms (shown in Fig. S5†). Experiments were either performed starting at 4 °C or 37 °C to obtain a population of tubulin in the free, unpolymerized state (4 °C) or polymerized state as MTs (37 °C), as these two structures present a different collection of binding sites to the ligand.

**Table 2:** Thermodynamic binding data for **RPC2**, DTX, and colchicine with tubulin and preformed microtubules as determined by ITC

Complex	Ligand	*K* (M^−1^) × 10^−6^	Δ*G* (kcal mol^−1^)	Δ*H* (kcal mol^−1^)	−*T*Δ*S* (kcal mol^−1^)
Tubulin	DTX	8.1	−9.4 ± 1.0	−28.7 ± 4.1	19.2
MT	DTX	5.5	−9.2 ± 0.9	−33.4 ± 6.5	24.7
MT	**RPC2**	4.8	−9.1 ± 0.9	−16.6 ± 2.3	7.5
MT	Colchicine	5.0	−9.1 ± 0.9	−12.8 ± 2.4	3.7
MT:DTX	**RPC2**	2.0	−8.6 ± 0.9	−14.6 ± 7.7	6.0

As seen from Table 2, **RPC2** binds to preformed MTs tightly, with a *K* of 4.9 × 10^6^ M^−1^ which is nearly the same as that obtained for DTX binding the preformed MTs (*K* = 5.5 × 10^6^ M^−1^) for DTX binding to preformed MTs. Reported association constants PTX and DTX with MTs are 10.7 × 10^6^ M^−1^ and 30.9 × 10^6^ M^−1^, respectively,^54,55^ which is in excellent agreement with our data given that different techniques were used (ITC *vs.* competitive binding titrations).^56^ A binding stoichiometry of one ligand per tubulin dimer is known for DTX and colchicine, which was also supported for **RPC2** from the inflection point of the isotherms at a 1 : 1 mole ratio. ITC experiments measuring the binding heat of **RPC2** to free tubulin showed excessive heat, consistent with the induction of MT polymerization and preventing any meaningful interpretation of the binding heat. ITC experiments injecting free tubulin into colchicine produced insufficient heat to successfully determine the binding parameters.

The differences in enthalpies and entropies of binding between **RPC2** and DTX show they have very different modes of binding and are very similar to those seen for colchicine. In competition experiments, the MT:DTX complex binds **RPC2** with the same energetics as MTs do, suggesting **RPC2** binds somewhere beside the taxane-binding domain.^8^

## Discussion

**RPC2** binds MTs *in vitro* and in live cells and this binding retards or arrests MT growth in a manner similar to that seen for PTX. As MSA activity is observed for **RPC2***in vitro*, the presumption is that the same is occurring *in vivo*, which is most strongly supported by the movie data, as the interpretation of the MT structure in fixed cells is often ambiguous. In MCF7 cells, the fast-moving and starburst pattern of MT comets characteristic of normal MT plus end growth is replaced by fewer, but larger comets that move slowly or are completely arrested by 2 h of treatment. Moreover, the organization of the MT growth by MOCs becomes less apparent and even absent in some cases. PTX is known to reduce the free tubulin concentration in cells to below a critical threshold necessary for organization by MOCs,^44^ and the western blot data in Fig. 4 and movie data show **RPC2** behaves in the same manner. A review of how simple divalent cations affect MT formation and depolymerization shows that divalent cations, such as Ca^2+^, Pb^2+^ and Hg^2+^, generally act to depolymerize or destabilize MTs.^12,13^ The MSA activity seen for **RPC2** contrasts with these simple cations, and shows the observed effect is not simply a charge effect.

Flow cytometry of MCF7 or H358 cells stained with PI showed only a small rise in G2/M population upon treatment with **RPC2** and this required a 17 μM dose, as the IC_50_ dose (1.5 μM) showed no effect. By comparison, the population in G2/M for PTX (0.25 μM) treated mouse fibroblast cells is ∼50% after 9 h and ∼100% by 27 h.^57^ The IC_50_ for PTX in this cell line is 0.86 μM.^58^ Apparently, **RPC2** is functioning in some other manner beside the mitotic arrest mechanism common for many MSAs. G2/M block is not the only mechanism by which MSA's, including PTX, function as the formation of aberrant MT structures (typically bundles) throughout the cell cycle; often implicated in cell death.^59–61^**RPC2** has also been shown to induce rapid depolarization of the mitochondria in A549 cells (within 2 h of administration), but this activity was not matched with an equal loss in cell viability, with 44% of the cells still alive 24 h later and the cell death was attributed to necrosis.^23^ The disconnect between time of mitochondrial depolarization and cell death was observed as an oddity by the authors of this report. Our findings and Glazers data support that **RPC2** was also acting *via* microtubule disruption, which alone or in concert with the mitochondrial effects is responsible for cell death.

ITC binding data reveals the strength of the binding for **RPC2** is comparable with DTX in preformed MTs and indicates that the binding site is not the taxane site. The extensive intracellular colocalization of **RPC2** with the cytoskeletal proteins reveals this selective binding is occurring in the cellular milieu. Our subcellular localization data conflicts with that reported by Glazer and coworkers in which **RPC2** was shown to co-localize with mitochondria and lysosomes.^23^ Using the inherent luminescence of **RPC2**, confocal luminescence microscopy experiments with additional organelles specific dyes, Mitotracker Green or Lysotracker Green, showed colocalization with these two cellular organelles. The phosphorescence of **RPC2** was also used by Audi *et al*. in microscopy experiments shows co-localization of **RPC2** with the nucleus in MDA-MB231 cells, however some localization at the cell membrane is also apparent. Part of this discrepancy is likely due to the fact that microtubules are only tens of nanometers thick and the binding of **RPC2** to them is diffuse through most of the cytoplasm, making it extremely hard to resolve at the light microscopy level. Secondly, microtubules are closely associated and more densely packed around the mitochondria and lysosomes, than for most other organelles^62,63^ and therefore part of the apparent discrepancy in co-localization data (Glazer data supports localization in the mitochondria and lysosomes whereas our data support co-localization in the cytoskeleton) is due to this overlap.

To date, there are no reports of RPCs or any coordination complexes acting selectively on the cytoskeleton *in vivo* or on cytoskeletal proteins *in vitro*. While the potent cytotoxicity of **RPC2** has been known for decades,^21–23^ the mechanism of action was either unknown or attributed to mitochondrial poisoning. Other cytotoxic RPCs are known and more are being developed as the field grows, but until this report the only cellular targets implicated have been the nuclear DNA, mitochondria, cell membrane, endoplasmic reticulum, and ribosomes.^30,32,64,65^ While not all RPCs share the three-bladed propeller structural motif, this trischelate structure is the most commonly explored motif to date in terms of biological probes, cellular interactions, and cytotoxicity.

From our cellular uptake data, we can infer that majority of the cytotoxicity of **RPC2** over **RPC1** is due to the enhanced uptake (approximately 10 fold greater) by the more lipophilic complex (log *P* 1.4 and −1.5, respectively^66^). The differential levels of colocalization with the cytoskeletal proteins, 82% for **RPC2** and 23% **RPC1**, reveals that the necessity of the DIP ligand over phenanthroline for tight binding. It is unknown if one, two or all three ligands must be DIP, but a review of the cytotoxicity of seven other RPCs containing at least 2 DIP ligands, *i.e.* [(DIP)_2_Ru(L-L)]^2+^ (where L-L is a chelating diamine), show IC_50_'s ranging from 4 to 11 μM against B16, SF, ML2, 4T1 or MCF7 cell lines.^20,21,67^ This high potency is suggestive that these other RPCs are also acting as MSAs in cells and the ‘Ru(DIP)_2_’ fragment is all that is needed for activity. We do not observe significant differences in the IC_50_'s for malignant *versus* non-malignant cell lines for **RPC2** and most other RPCs with a ‘Ru(DIP)_2_’ fragment behave similarly (when such data is available). Two important exceptions are [(DIP)_2_Ru(tatpp)]^2+^ and [(DIP)_2_Ru(tatpp)Ru(DIP)_2_]^2+^ (tatpp = tetraazatetrapyridopentacene), which show selectivity indices (SI = IC_50_ MCF10/IC_50_ MCF7) of 71 and 11, respectively.^20^

There are no obvious correlations between the structure of **RPC2** and the structures of other natural product MSA's (and derivatives thereof). Natural product MSA's tend to be more cytotoxic with IC_50_'s in the low micromolar to nanomolar regime where as **RPC2** is in the low micromolar regime. Most of the natural products exhibit very complex organic structures containing multiple fused rings, numerous chiral centers, and require challenging multi-step syntheses.^68^ Attractively, RPCs are relatively simple and can be prepared in one to three steps from the reaction of the commercially available ligands with a Ru salt.^22,24,25,69,70^ We prepared over 2 g of **RPC2** using two steps by refluxing RuCl_3_ and excess DIP in ethanol, isolating the hexafluorophosphate salt and metathesis to the chloride salt. Overall yield was 83% and it took less than 2 days. **RPC2** has recently become commercially available (*e.g.* Sigma-Aldrich, Alfa Aesar, Spectrum Chemical), as a fluorescent dye. It should be noted that **RPC2** is chiral at the metal ion (*D*_3_ point group symmetry) and is typically isolated as the racemate, but can be resolved to stable enantiomers.^71^ This newly reported MSA activity for **RPC2** and their relative ease of preparation and derivatization suggests this class of compounds are ripe for exploration in this new application.

## Conclusions

The combination of the live cell movies, fixed cell MT structure, cytotoxicity, sub-cellular localization, *in vitro* MT binding, polymerization, and TEM data build a convincing case that **RPC2** enters cells, binds, and disrupts the normal MT network by stabilizing the MTs present. **RPC2** exhibits a reasonably high binding constant with preformed MTs which does not appear to be the taxane binding site favored by many MSAs. *In vitro* polymerization assays show **RPC1** can also promote MT formation, suggesting that RPCs in general can act as MSAs (to different degrees) and that some of the DIP containing RPCs, previously explored as cytotoxins are actually targeting MTs. This is the first report of any transition metal complex or organometallic complex demonstrating MT binding and disruption of MT function. While much remains to be elucidated about the MSA activity of this and related compounds, these data show that RPCs with proper ligand modifications are likely to constitute a new, synthetically accessible, class of compounds that can be used to target MTs in cells, with potentially therapeutic benefits *in vivo*.

## Conflicts of interest

There are no conflicts to declare.

## Supplementary Material

SC-011-C9SC05671H-s001

SC-011-C9SC05671H-s002
