# Supplementary material for: Disruption of microtubule function in cultured human cells by a cytotoxic ruthenium(ii) polypyridyl complex
Source: Chem Sci. 2019 Nov 18;11(1):264–75. doi: 10.1039/c9sc05671h (PMC8133002; doi:10.1039/c9sc05671h)
Supplement: SC-011-C9SC05671H-s001 [file SC-011-C9SC05671H-s001.pdf]

Electronic Supplementary Material (ESI) for Chemical Science.
This journal is © The Royal Society of Chemistry 2019

Supporting Information

Disruption of Microtubule Function in Cultured Human Cells by a Cytotoxic Ruthenium(II) Polypyridyl Complex

Nagham Alatrash[1], Faiza H. Issa[1], Nada S. Bawazir[2], Savannah West[3], Kathleen E. Van Manen-Brush[2], Charles P. Shelor[1], Adam S. Dayoub[1], Kenneth A. Myers[2], Christopher Janetopoulos[2], Ed Lewis[3], and Frederick M. MacDonnell[1*]

[1]Department of Chemistry and Biochemistry, University of Texas at Arlington, Arlington, TX, 76019

[2] Department of Biological Sciences, University of the Sciences, Philadelphia, PA 19104

[3] Department of Chemistry, Mississippi State University, Starkville, MS 39762

Supporting Figures **S2**

Figure S1. **p. S4**

Spinning disk confocal fluorescent microscopic images of live MCF7 and MCF10a cells expressing the GFP–EB. Images show the initial frame of a short movie (4-9 s) and changes between images are due to the longer incubation period with 1.7 μM RPC2 or 0.1 μM PTX for MCF7 and 0.2 μM PTX for MCF 10a. *This is a PowerPoint file and does not work in other formats* ***(separate file).*** *Details about the individual movies are on page s31.*

Figure S2. **p. S6**

Effect of treatment with RPC2 for 12 h on the cell cycle distribution in H358 breast cancer cells. RPC2 affects the cell cycle distribution in H358 breast cancer cells. H358 cells were untreated (A) or treated with IC_{50} of 17 μM RPC2 for 12 h, fixed with 66% ethanol then washed in PBS and treated with RNase for 30 min at 37°C and stained with PI. Control distribution: SubG 7%, G1 68%, S 13%, G2/M 12%. RPC2 treated: Sub G 8%, G1 63%, S 10%, G2/M 19%

Figure S3. **p. S7**

In vitro dose dependent changes in the polymerization rate and extent of polymerization for tubulin treated with RPC2. The change in turbidity measured by light transmission at 340 nm. Increasing turbidity indicated tubulin polymerization upon a temperature jump from 4°C to 37°C in the presence of 1 mM GTP and 10% glycerol in general tubulin buffer (80 mM PIPES pH 6.9, 2 mM $MgCl_2$, and 0.5 mM EGTA). All runs with added drug were done by addition of different concentration between 0.1-50 μM solution of drug with the tubulin (3 mg/mL). The dose of RPC2 is indicated in the key above the figure.

Figure S4. **p. S8**

Transmission electron micrographs of freshly formed microtubules after depositing on 300-mesh carbon-coated, formvar-treated copper grids and stained with 1% (w/v) uranyl acetate.

Top row: Control, NCZ (10 μM) and PTX (10 μM)

Bottom row: Three images of MTs formed in the presence RPC2 (10 μM). The samples were stained. The grids were examined in a Zeiss model 10CA electron microscope. The scale bar is 100 nm.

Figure S5. **p. S9**

Reverse titration integrated isotherms for the binding or ligands (RPC2, DTX, or colchicine) with tubulin or preformed microtubules. Red lines show the nonlinear regression fits of the ITC integrated heat data for the reverse titration of tubulin or microtubules into solutions of DTX, RPC2, or Colchicine. Experiments with tubulin were performed at 4°C and those with MTs at 37°C. The fits shown were used to obtain the data in Table 1.

Figure S1. This is a separate Powerpoint file containing 20 avi files. The movies are labeled 1 through 20 starting down the first column (M1-M5) second column (M6-M10) third column (M11-M15), and forth column (M16-M20).

Details for individual movies. Each Movie is also provided as a separate avi file in the SI

Movie S1. Spinning disk confocal microscopy video of live MCF7 expressing the GFP-EB3. Video taken before incubation with 1.7 μM RPC2. Time frames taken every 2 seconds.

Movie S2. Spinning disk confocal microscopy video of live MCF7 expressing the GFP-EB3. Video taken after 30 minutes incubation with 1.7 μM RPC2. Time frames taken every 2 seconds.

Movie S3. Spinning disk confocal microscopy video of live MCF7 expressing the GFP-EB3. Video taken 1 hour after incubation with 1.7 μM RPC2. Time frames taken every 2 seconds.

Movie S4. Spinning disk confocal microscopy video of live MCF7 expressing the GFP-EB3. Video taken 2 hours after incubation with 1.7 μM RPC2. Time frames taken every 2 seconds.

Movie S5. Spinning disk confocal microscopy video of live MCF7 expressing the GFP-EB3. Video taken after 3 hours incubation with 1.7 μM RPC2. Time frames taken every 2 seconds.

Movie S6. Spinning disk confocal microscopy video of live MCF10a cells expressing GFP-EB3. Video taken before the incubation with 1.7 μM RPC2. Time frames taken every 2 seconds.

Movie S7. Spinning disk confocal microscopy video of live MCF10a cells expressing GFP-EB3. Video taken after 30 minutes incubation with 1.7 μM RPC2. Time frames taken every 2 seconds.

Movie S8. Spinning disk confocal microscopy video of live MCF10a cells expressing GFP-EB3. Video taken after 1 hour incubation with 1.7 μM RPC2. Time frames taken every 2 seconds.

Movie S9. Spinning disk confocal microscopy video of live MCF10a cells expressing GFP-EB3. Video taken after 2 hours incubation with 1.7 μM RPC2. Time frames taken every 2 seconds.

Movie S10. Spinning disk confocal microscopy video of live MCF10a cells expressing GFP-EB3. Video taken after 3 hours incubation with 1.7 μM RPC2. Time frames taken every 2 seconds.

Movie S11. Spinning disk confocal microscopy video of live MCF7 cells expressing GFP-EB3. Video taken before incubation with 0.1 μM PTX. Time frames taken every 2 seconds.

Movie S12. Spinning disk confocal microscopy video of live MCF7 cells expressing GFP-EB3. Video taken 30 minutes after incubation with 0.1 μM PTX. Time frames taken every 2 seconds.

Movie S13. Spinning disk confocal microscopy video of live MCF7 cells expressing GFP-EB3. Video taken after 1 hour incubation with 0.1 μM PTX. Time frames taken every 2 seconds.

Movie S14. Spinning disk confocal microscopy video of live MCF7 cells expressing GFP-EB3. Video taken after 2 hours incubation with 0.1 μM PTX. Time frames taken every 2 seconds.

Movie S15. Spinning disk confocal microscopy video of live MCF7 cells expressing GFP-EB3. Video taken 3 hours incubation with 0.1 μM PTX. Time frames taken every 2 seconds.

Movie S16. Spinning disk confocal microscopy video of live MCF10a cells expressing GFP-EB3. Video taken before the incubation with 0.2 μM PTX. Time frames were taken every 2 seconds.

Movie S17. Spinning disk confocal microscopy video of live MCF10a cells expressing GFP-EB3. Video taken 30 minutes after incubation with 0.2 μM PTX. Time frames taken every 2 seconds.

Movie S18. Spinning disk confocal microscopy video of live MCF10a cells expressing GFP-EB3. Video taken after 1 hour incubation with 0.2 μM PTX. Time frames taken every 2 seconds.

Movie S19. Spinning disk confocal microscopy video of live MCF10a cells expressing GFP-EB3. Video taken after 2 hours incubation with 0.2 μM PTX. Time frames taken every 2 seconds.

Movie S20. Spinning disk confocal microscopy video of live MCF10a cells expressing GFP-EB3. Video taken after 3 hours incubation with 0.2 μM PTX. Time frames taken every 2 seconds.

Figure S2.

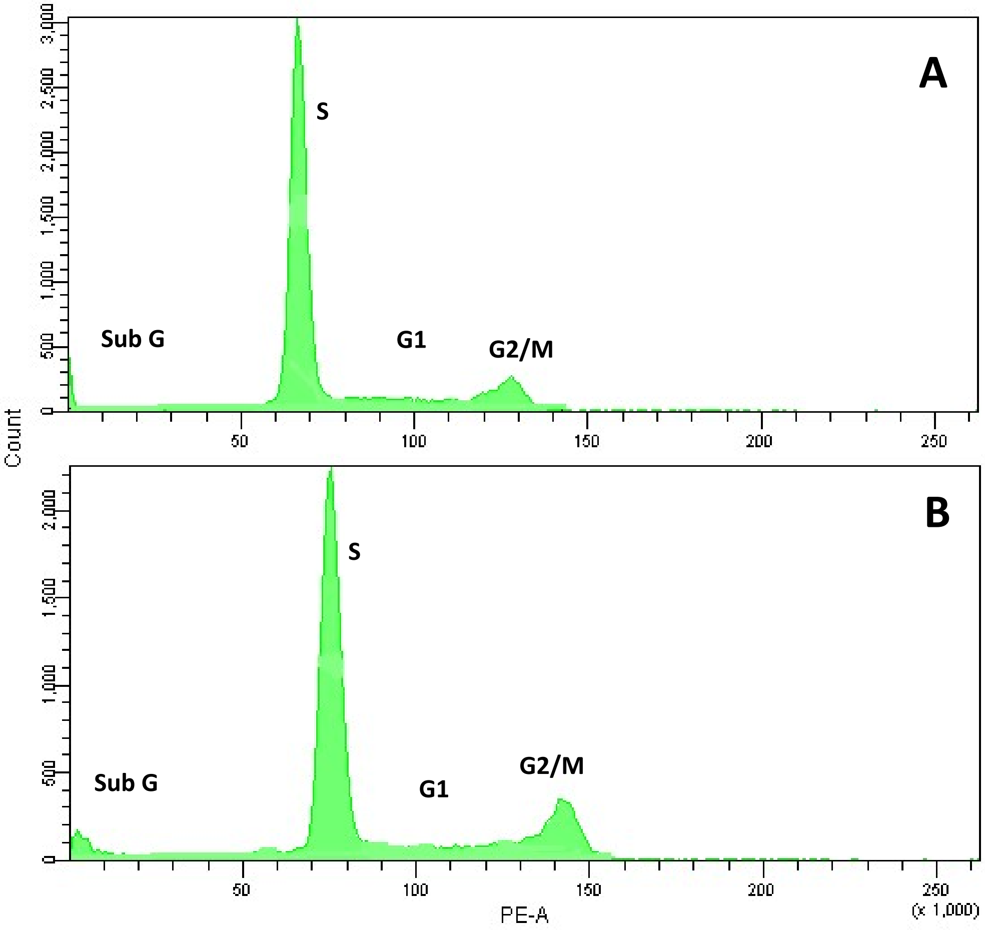

Figure S3.

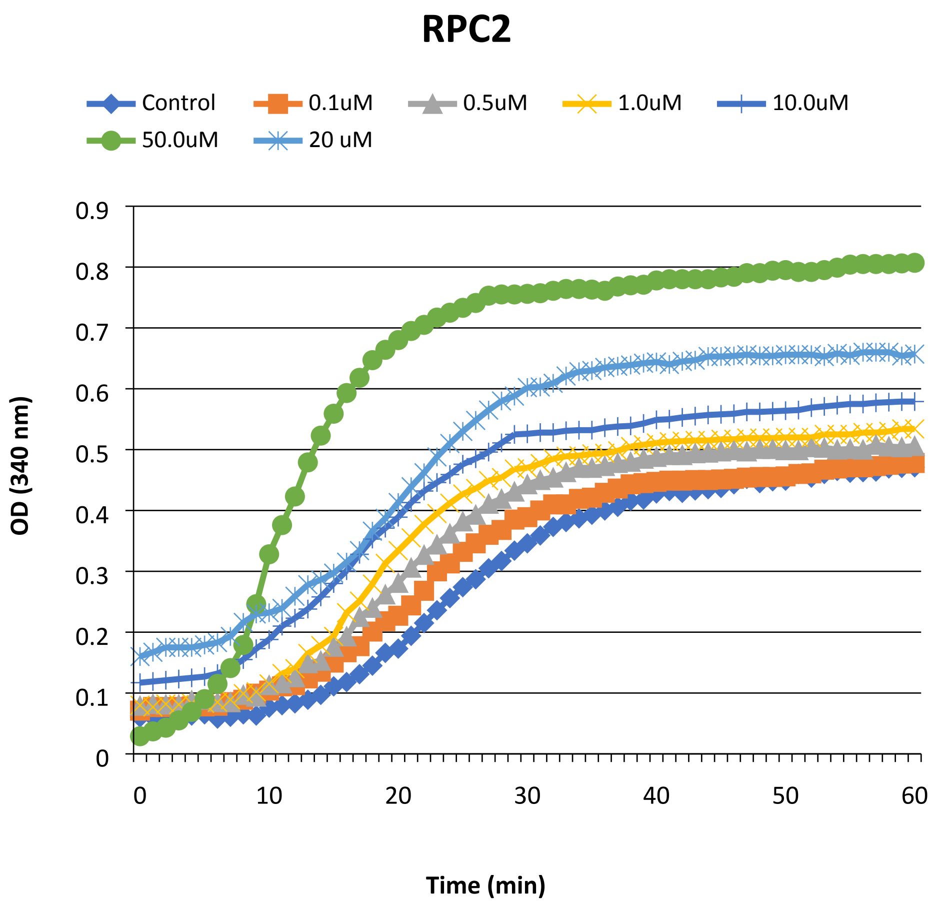

RPC2
Control
0.1uM
0.5uM
1.0uM
10.0uM
50.0uM
20 uM
OD (340 nm)
0.9
0.8
0.7
0.6
0.5
0.4
0.3
0.2
0.1
0
0
10
20
30
40
50
60
Time (min)

Figure S4.

Control (vehicle only) **NCZ (10 μM)** **PTX (10 μM)**

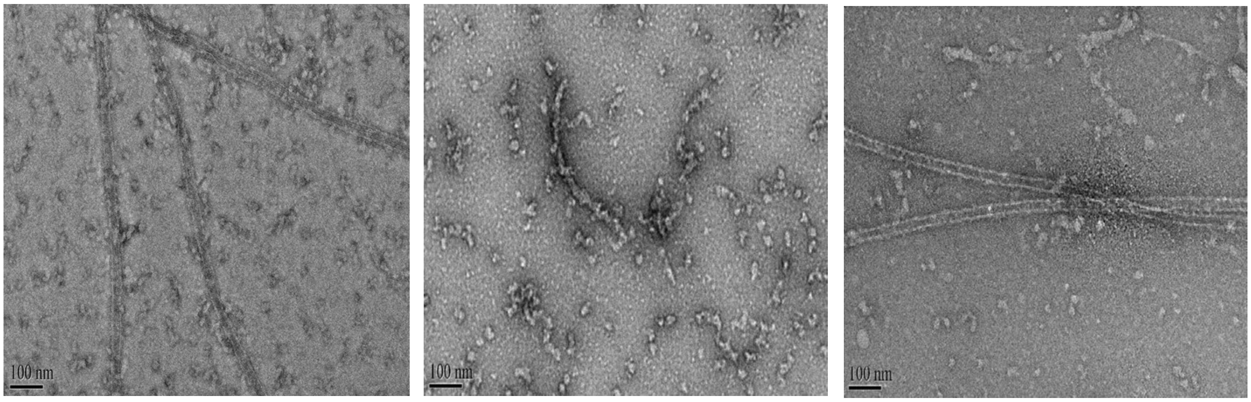

All three images below are for MTs formed in the presence of RPC2 (10 μM)

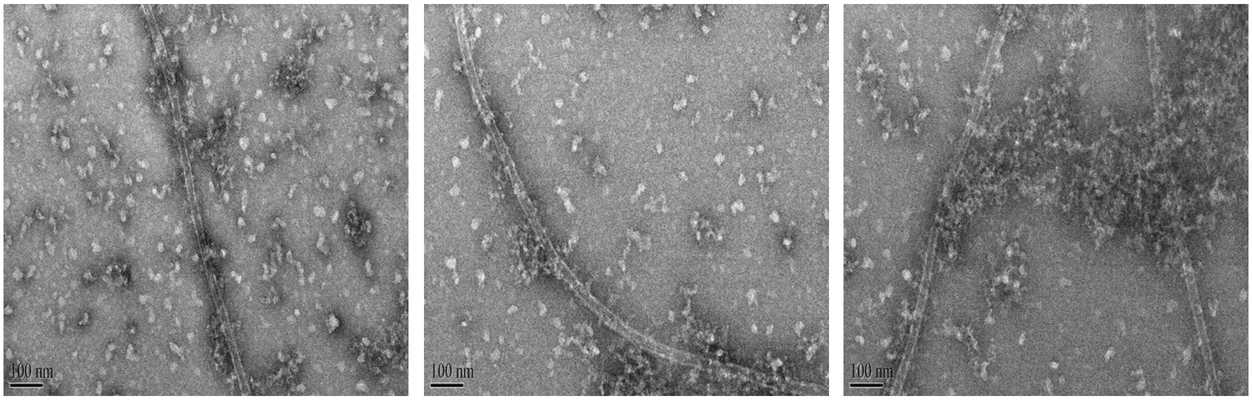

Figure S5.

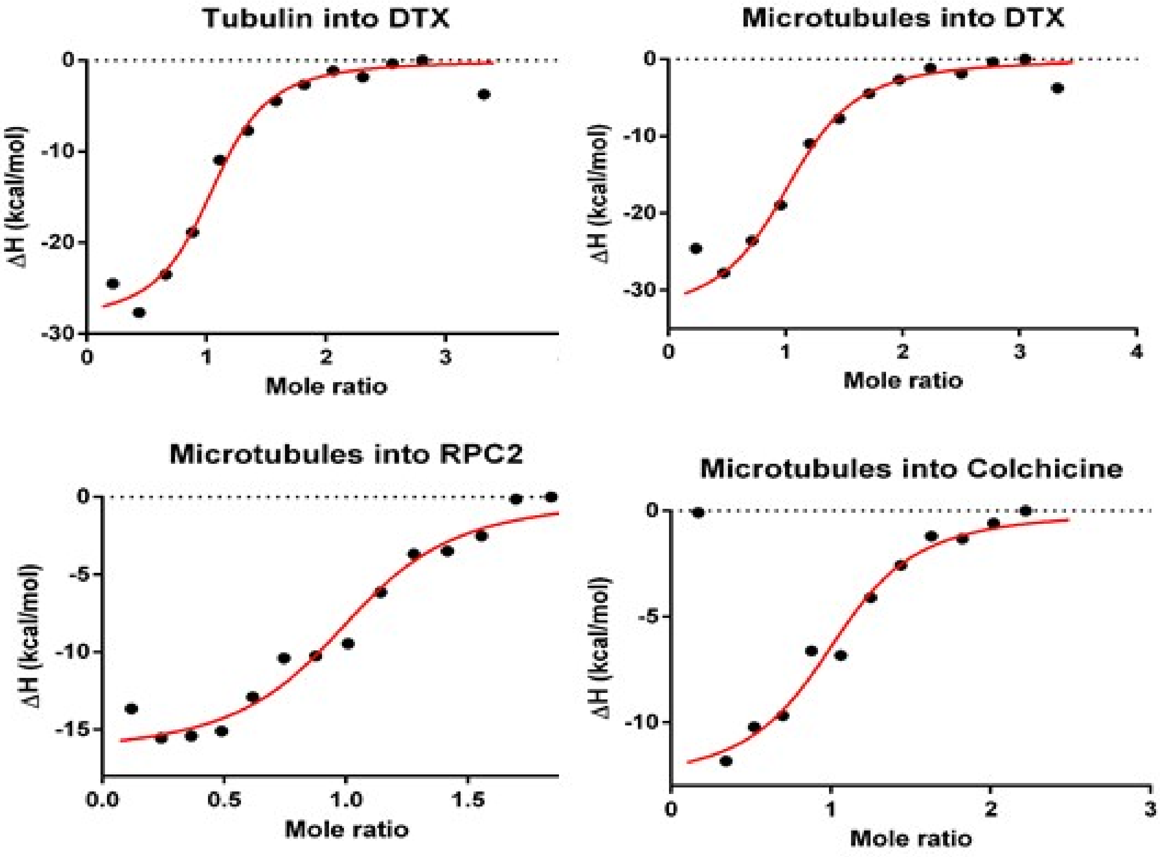

Tubulin into DTX
ΔH (kcal/mol)
0
-10
-20
-30
0
1
2
3
Mole ratio
Microtubules into DTX
ΔH (kcal/mol)
0
-10
-20
-30
0
1
2
3
4
Mole ratio
Microtubules into RPC2
ΔH (kcal/mol)
0
-5
-10
-15
0.0
0.5
1.0
1.5
Mole ratio
Microtubules into Colchicine
ΔH (kcal/mol)
0
-5
-10
0
1
2
3
Mole ratio
