# Supplementary material for: Disruption of microtubule function in cultured human cells by a cytotoxic ruthenium(ii) polypyridyl complex
Source: Chem Sci. 2019 Nov 18;11(1):264–75. doi: 10.1039/c9sc05671h (PMC8133002; doi:10.1039/c9sc05671h)
Supplement: SC-011-C9SC05671H-s002 [file SC-011-C9SC05671H-s002.pptx]

## Slide 1
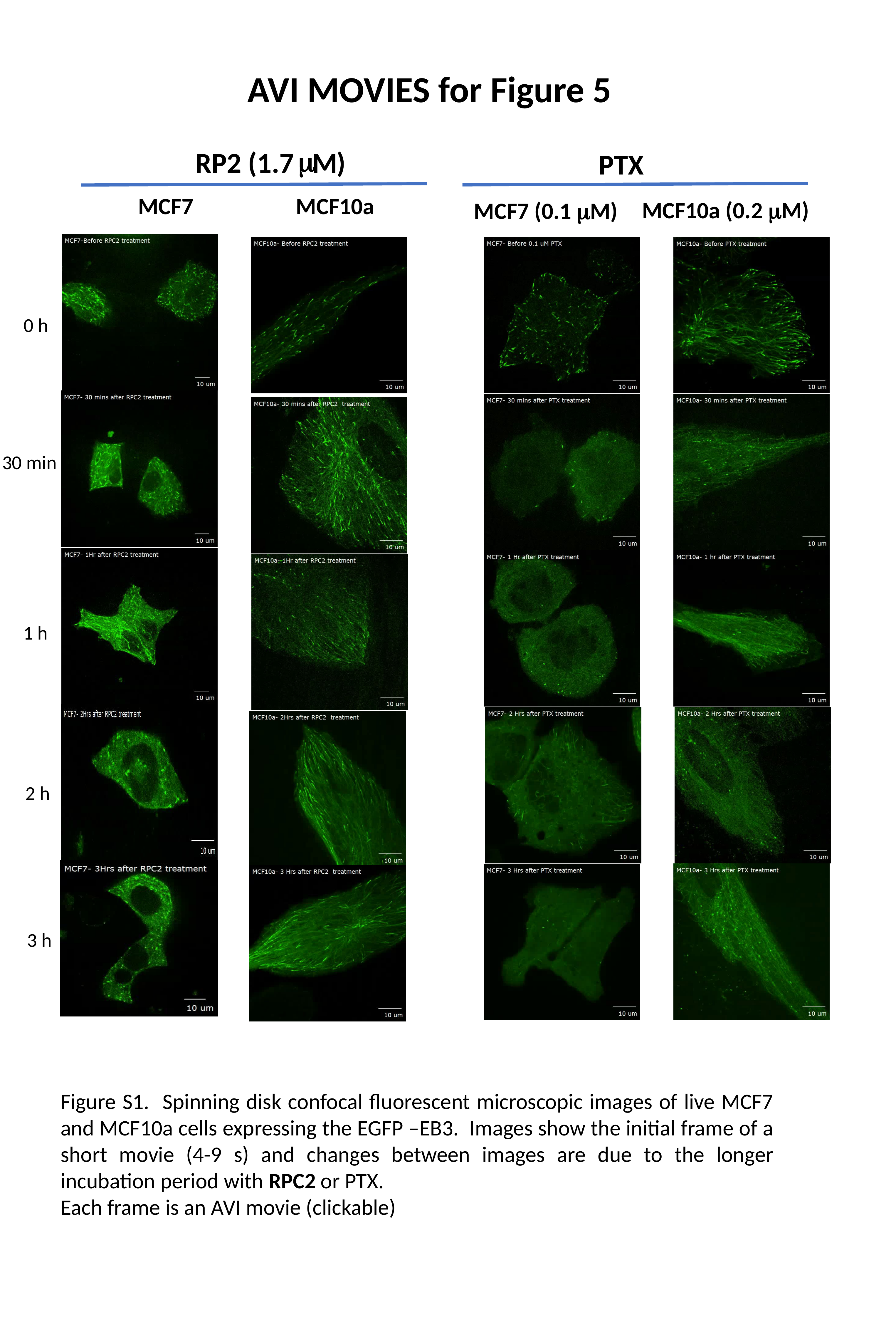

AVI MOVIES for Figure 5
m
RP2 (1.7
M
)
PTX
MCF7
MCF10a
MCF10a (0.2 mM)
MCF7 (0.1 mM)
0 h
30 min
1 h
2 h
3 h
Figure S1. Spinning disk confocal fluorescent microscopic images of live MCF7 and MCF10a cells expressing the EGFP –EB3. Images show the initial frame of a short movie (4-9 s) and changes between images are due to the longer incubation period with RPC2 or PTX.
Each frame is an AVI movie (clickable)
